# Oral intake management in laboring women: a scoping review

**DOI:** 10.3389/fmed.2025.1690743

**Published:** 2025-10-28

**Authors:** Chenping Zhu, Lin Zhou, Yongjie Tang

**Affiliations:** ^1^School of Nursing, Zhejiang Chinese Medical University, Hangzhou, China; ^2^Department of Nursing, Hangzhou First People's Hospital, Hangzhou, China

**Keywords:** oral intake, labor, parturient, obstetric, scoping review

## Abstract

**Background and objectives:**

Labor is a physically demanding and painful process that may lead to fat breakdown, ketone accumulation, and ketosis, potentially resulting in metabolic acidosis. Proper management of oral intake during labor helps mitigate this risk. We reviewed the published impact of oral intake management during labor on maternal and neonatal outcomes.

**Methods and study design:**

The scoping review used Arksey and O′Malley’s methodological framework. The systematic search was conducted using PubMed, Web of Science, Embase, Cochrane Library, Scopus, CNKI, and CINAHL Complete databases in May 2025. The literature published in the database until May 2025 was searched.

**Results:**

A total of 17 studies, involving 13,141 participants, were included in this review. Out of these, 15 studies were randomized controlled trials, and 2 were observational studies. The participants in this review were low-risk parturients without maternal illness. The oral intake during labor included carbohydrate-rich beverages, isotonic sports drinks, high-protein drinks, bicarbonate solutions, and other similar beverages. The outcomes of this review encompassed both maternal and neonatal outcomes. The review did not identify any significant harms associated with moderate oral intake.

**Conclusion:**

Moderate oral intake did not prolong labor duration in low-risk parturients and helped maintain energy expenditure during labor. It also stabilized blood glucose and electrolyte levels, preventing maternal hypoglycemia and ketoacidosis. However, due to concerns about aspiration and labor progress, a multidisciplinary approach and individualized dietary plan were essential to optimize the type and timing of intake. Generally, light carbohydrate diets were recommended in the early stages of labor, while high-protein and isotonic energy drinks might be more suitable during the second stage for low-risk women.

**Systematic review registration:**

https://osf.io/vmahc/overview.

## 1 Introduction

Labor is a period of significant fluid and energy loss, which may lead to increased pain and negative birth experiences. Insufficient energy intake during labor can result in maternal hypoglycemia and increased lipolysis, which in turn promotes fatty acid oxidation and ketone body production (1). These metabolic changes have a certain probability of causing prolonged labor, increased cesarean section rates, neonatal hypoglycemia, metabolic acidosis, and other adverse outcomes (2). Although intravenous fluids are not recommended for preventing ketosis generally due to potential negative metabolic and physiological effects on both mother and fetus (3). Parturients remain awake during labor and are capable of expressing their own will; excessive dietary restrictions can lead to emotional distress, such as unhappiness and stress (4).

In 1997, the World Health Organization reviewed existing data and recommended that healthcare providers respect a woman’s desire for oral intake during labor, given the potential benefits for both maternal and neonatal health (5). Proper oral intake —such as carbohydrates and isotonic solutions—can stabilize blood glucose and electrolyte levels, reduce the risk of hypokalemia, and shorten labor duration (6). Additionally, it has been shown to reduce labor fatigue and support a synergistic effect between mind and body (7).

In contrast, a 2007 guideline from the American Society of Anesthesiologists recommended avoiding solid food during labor, citing an increased risk of maternal complications (8). They also recommended that modest amounts of clear liquids—such as water, clear tea, black coffee, and sports drinks—may be appropriate for uncomplicated parturients. For those with risk factors for aspiration (e.g., morbid obesity, diabetes, or difficult airways), further restrictions on oral intake should be considered on a case-by-case basis.

Oral intake during labor can include a variety of options, such as carbohydrates, sugar-sweetened beverages, high-protein drinks, nutritional supplements, and energy beverages. Despite several studies trying to identify optimal foods and drinks, no consensus has been reached regarding the best approach to supplementation. In the Yangtze River Delta region of China, for example, 18.8 and 6.8% of the parturients were not informed about the appropriate intake of solid food and non-clear liquids during labor, highlighting the lack of standardization in oral intake management (9).

Previous studies have used meta-analyses to examine the effects of oral intake interventions during labor. Malin et al. conducted a meta-analysis on oral carbohydrate supplementation and found that small amounts of carbohydrate did not significantly impact labor outcomes (10). Salajegheh et al. demonstrated that consuming dates in the peripartum period significantly shortened the labor length (11). But these studies primarily focused on individual methods of oral intake, and comprehensive analyses that synthesize multiple types of oral nutrition were limited or lacking. Scoping reviews were particularly helpful when the literature was complex and heterogeneous (12).

Given the variability in practices and the lack of clear guidelines, this scoping review aims to synthesize current approaches to oral intake management during labor, evaluate its impact on birth outcomes, and provide a reference for future research.

## 2 Methods

### 2.1 Protocol development

The methodology for this scoping review was adapted from Arksey and O′Malley’s framework (13). This approach involves five stages: (1) identifying the research questions; (2) identifying relevant studies; (3) study selection; (4) charting and collating data; (5) summarizing and reporting information. This scoping review adhered to the Preferred Reporting Items for Systematic Reviews and Meta-Analyses Extension for Scoping Reviews (PRISMA-ScR). The final protocol was registered prospectively with the Open Science Framework on December 22, 2024^1^.

### 2.2 Stage 1: identifying the research question

We used the PICOS (Population, Intervention, Comparison, Outcome, Study design) framework to formulate the research question. P (population): low-risk parturients; I (intervention): oral intake management during labor; C (comparison intervention): control or alternative groups; O (outcome): maternal and neonatal outcomes; S (Study design): quantitative studies. Therefore, the research question identified was: “What is the effect of oral intake management during labor on maternal and neonatal outcomes compared to control or alternative groups?”

### 2.3 Stage 2: identifying the relevant studies

A systematic search was conducted across electronic databases, using standardized search terms tailored to the needs of each database and refined by the research team. The search covered all available records from the inception of the databases through May 2025. The databases included in the search were PubMed, Web of Science, Embase, Cochrane Library, Scopus, CNKI, CINAHL Complete databases. The following combined relevant Medical Subject Headings terms and keywords were used:

#1: (((((oral intake) OR (energy management)) OR (energy)) OR (diet)) OR (food)) OR (drink).

#2: (((((((((((carbohydrate) OR (protein)) OR (ice chips)) OR (fat)) OR (solid food)) OR (energy drink)) OR (sugary drink)) OR (milk)) OR (water)) OR (chocolate)) OR (candy)) OR (bread).

#3: (maternal outcomes) OR (delivery outcome).

#4: (((((((((duration of labor) OR (intrapartum blood loss)) OR (cesarean section rate)) OR (aspiration)) OR (childbirth satisfaction)) OR (aspiration)) OR (nausea)) OR (emesis)) OR (apgar score)) OR (hypoglycaemia).

#5: (((labor) OR (childbirth)) OR (parturition)) OR (intrapartum).

#6: #1 AND #2 AND #3 AND #4 AND #5.

### 2.4 Stage 3: selecting studies

*The candidate studies should be eligible for inclusion based on the following inclusion criteria:* (a) studies involving low-risk women presenting with 36 + weeks spontaneous cephalic labor; (b) studies involving oral intake management during labor; (c) studies published in English or Chinese. *Studies met following criteria were excluded*: (a) studies involving planned cesarean section; (b) studies involving maternal illness such as diabetes, hypertension, or prior gastric/esophageal surgery; (c) studies involving intravenous therapy; (d) review studies, comments, letters, meta-analysis; (e) Studies that were reviews, qualitative studies, conference abstracts, case reports, protocols, or focused on unrelated topics.

Abstracts that did not meet the inclusion criteria or fell under the exclusion categories were discarded, and were restricted to English or Chinese publications. Duplicate records across databases were removed using EndNote X7 for Windows. Screening occurred in two stages: initially, titles and abstracts were reviewed, followed by full-text screening. Two independent reviewers assessed the records for inclusion. A third reviewer solved any disagreements.

### 2.5 Stage 4: charting and collating data

To confirm the relevance of the studies and extract their characteristics, we analyzed the selected studies using a standardized system designed for this scoping review. The following information was obtained: first author, publication year, country, study design methods, sample size, content of oral intake management, timing of intervention, and primary outcomes.

### 2.6 Stage 5: summarizing and reporting results

The extracted data from the included studies were summarized. This review followed a scoping review methodology, with no need to evaluate the quality of the evidence (11). So the key characteristics were analyzed without critical appraisal.

## 3 Results

### 3.1 Study selection

**Figure 1:**
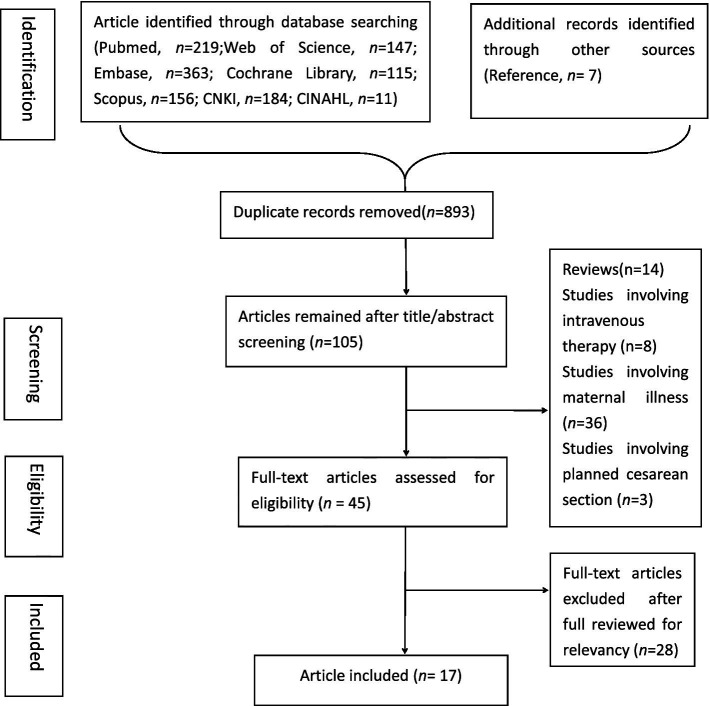
Flowchart of literature searching.

The search strategy identified 1,195 studies, with seven additional articles found through reference list scanning. After removing duplicates, 893 articles remained. Following title and abstract screening, 788 articles were excluded, leaving 44 studies for full-text evaluation. Ultimately, 17 studies, comprising 13,141 participants, were included in the scoping review. The selection process is illustrated in Figure 1.

### 3.2 Study characteristics

The study characteristics were summarized in Table 1. A total of 13,141 women were included across the selected studies, which were published between 1999 and 2025. The studies included laboring women from nine countries: the United States, the United Kingdom, France, the Netherlands, Iran, Israel, Norway, China, and India. All participants were healthy, with no maternal illnesses such as diabetes mellitus, hypertension, previous gastric or esophageal surgery, or contraindications to vaginal delivery. Of the 17 studies, 15 were randomized controlled trials, and two were observational studies.

**Table 1:** Characteristics of the included studies.

Author, year, country	Study design methods	Samples (T/C)	Content of oral intake management in T	Content of oral intake management in C	Timing of intervention	Primary outcomes	Data source
Maor GS et al., 2024, Israel (27)	RCT	129 (58/71)	Not limited to specific foods, but recommended avoiding greasy food and suggested a diet of light food (energy bars, fruit or yogurt)	clear liquids only	Consume food at least every 2 h during the end of the latent phase of the early second phase of labor	(a) + ↓: T had a significantly shorter duration of the second stage of labor; (f) −: 5 min Apgar score	Pubmed
Rahmani R et al., 2023, Iran (20)	RCT	60 (30/30)	15 mL of date palm sap per hour	15 ml of sugar and water solution per hour	From the beginning of the active phase until its end	(a) + ↓: T had a significantly shorter duration of labor; (d)−: pain and anxiety score	Embase
Ting Ding et al, China (26)	RCT	1953 (982/971)	Carbohydrate-rich beverage (65 kcal/h)	Low-carbohydrate beverages (18 kcal/h)	During labor after initiating epidural labor analgesia	(b)−: rate of cesarean delivery; (d) + ↓: T had a significantly lower subjective hunger score	Pubmed
Mitra Seyedi et al., 2021, Iran (24)	RCT	142 (71/71)	200 mL bicarbonate solution (4.26 g sodium bicarbonate) with routine oral intake (water, juice, cakes, dates)	Routine oral intake including water, juice, cakes and dates	Onset of active labor (4 cm cervical dilation)	(a) + ↓: T had a significantly shorter duration of labor; (b) + ↓: T had a significantly lower rate of instrumental delivery (f)−: neonatal Apgar score	Pubmed
Kanneganti, Aishwarya, 2020, India (17)	Prospective observational study	211 (50/161)	Combination of caloric clear liquids (watermelon juice, apple juice, coconut water, iced tea, lime juice, clear soups) and solids (biscuits, bananas, sandwiches)	Calorific clear liquids only (watermelon juice, apple juice, coconut water, iced tea, lime juice, clear soups)	Throughout labor	(a) − duration of labor; (b) −: type of delivery; (c) −: gastric aspiration; (f) −: admission of neonate to NICU and APGAR scores	Web of science
Simonet, T. et al., 2020, France (14)	RCT	3,984 (2014/1970)	200 mL of apple or grape juice without pulp every 3 h	Fasting (water only)	During labor, < 8 cm of cervical dilatation	(a) −: duration of labor; (b) −: the rate of cesarean delivery; (c) + ↑: T had significantly more vomiting (d)-: self-reported maternal feeling	Pubmed
Rousset J. et al., 2020, France (15)	RCT	125 (62/63)	400 mL of apple juice within 90 min before full cervical dilation	Fasting	From the first stage of labor, starting at cervical dilation > 2 cm	(c) −: maternal gastric emptying; (d) −: maternal anxiety or pain level	Pubmed
Shea-Lewis, A. et al., 2018, United States (28)	Retrospective observational cross-sectional study	2,797 (1,198/1599)	Eat and drink as pleased	Ice chips	Throughout labor	(b) + ↓: T had significantly less likely to have unplanned cesarean section births; (f) −: Apgar scores	CINAHL
Vallejo, M. C. et al., 2013, United States (22)	RCT	150 (75/75)	325 mL of high-protein drink	Ice chips/water	Consume within 15 min	(c) −: incidences of nausea and emesis; (d) + ↑: T had significantly higher satisfaction scores; (f) −: 1 and 5 min neonatal Apgar scores	Pubmed
Rahmani, R. et al., 2012, Iran (16)	RCT	177 (87/90)	Three dates or 110 mL of orange juice	Fasting (water only)	Before the start of the active phase (cervical dilation 3–4 cm)	(a) + ↓: T had a significantly shorter duration of labor; (b) −: type of delivery; (c) −: frequency and volume of vomiting; (f)-: 1 and 5 min Apgar scores	Pubmed
Kordi M et al., 2010, Iran (21)	RCT	90 (45/45)	Honey-date syrup	Placebo	Starting at cervical dilation > 4 cm	(a) + ↓: T had a significantly shorter duration of labor	Embase
Kardel, K. R. et al., 2010, Norway (18)	RCT	213(111/102)	1 litre of isotonic energy-drink	Placebo	Start at cervical dilatation of 3 cm	(a) −: duration of labor; (b) −: rate of instrumental vaginal deliveries and cesarean sections; (f)-: Apgar score	Pubmed
O’Sullivan, G. et al., 2009, United Kingdom (29)	RCT	2,426 (1,219/ 1,207)	Low-fat, low-residue diet (bread, biscuits, vegetables, fruits, low-fat yogurt, soup, isotonic drinks, fruit juice)	Ice chips/water	Starting from cervical dilation < 6 cm	(a) −: duration of labor; (b) −: rate of instrumental vaginal delivery or cesarean delivery; (c)-: maternal vomiting; (f) −: Apgar score and admission to neonatal intensive care or special care units	Pubmed
Tranmer JE et al., 2005, United Kingdom (30)	RCT	328 (163/165)	A variety of drinks and carbohydrate snacks (e.g., toast, fruits, crackers)	Ice chips/water	Throughout labor	(b)−: cesarean section rate; (e) −: ketones of labors:	Pubmed
Scheepers, H. C. J. et al., 2004, Netherlands (25)	RCT	202 (100/102)	200 mL carbohydrate solution	Placebo	From the second stage of labor (8–10 cm cervical dilation)	(b) + ↓: T had a significantly lower rate of cesarean section; (f) −: neonatal outcome;	Pubmed
Kubli, M. et al., 2002, United Kingdom (19)	RCT	60 (30/30)	500 mL isotonic sports drink in the first hour, followed by another 500 mL every 3 to 4 h	Water only	In early labor (cervical dilation 5 cm)	(a) −: duration of labor; (b) −: mode of delivery; (c) −: incidence of vomiting and the volume vomited; (f)-: Apgar scores and umbilical artery and venous gases	Pubmed
Scrutton, M. J. L. et al., 1999, United Kingdom (31)	RCT	94 (48/46)	Light diet	Water only	Throughout labor	(a) −: duration of labor; (b) −: mode of delivery; (f) −: Apgar scores and umbilical artery and venous gases	Pubmed

RCT means randomized controlled studies. T is the test group, C is the control group. Primary outcomes: (a) Relate to duration of labor; (b) Relate to mode of assisted delivery; (c) Relate to gastrointestinal complications; (d) Relate to maternal perception of labor; (e) Parturient ketosis; (f) Relate to neonatal outcomes.

+: Statistically significant difference; −: No statistically significant difference. ↑: T > C; ↓: T < C.

### 3.3 Content of oral intake management

This review encompassed a wide variety of oral intake interventions during labor, focusing on fluids and light diets. Most studies included carbohydrate-based intakes such as dates, bread, biscuits, vegetables, fruits, low-fat yogurt, soup, or fruit juice.

Several studies examined fruit juice consumption, found that fruit juice intake did not affect the rate of instrumental delivery (14–16). In these studies, one study noted more parturients in the 200 mL of apple or grape juice without pulp every 3 h experienced more vomiting (17). This discrepancy may be also due to variations in the volume of carbohydrate and the timing of intake—either early or late in labor (14, 16, 17).

Two articles mentioned isotonic drinks, the intervention was also performed in early labor (18, 19). By measuring gastric antral cross-sectional area, Kubli, M. et al. (19) found isotonic drinks could reduce maternal ketosis in labor without increasing gastric volume and was not associated with any maternal or neonatal outcomes.

Dates, including honey-dates and palm-dates, were also evaluated. Oral dates intake did not change the frequency of vomiting and mode of labor, or increase any other adverse neonatal outcomes. What’s more, intake of dates during active labor could decrease the second stage of labor phase, which could reduce the time that parturients endure pain and anxiety (16, 20, 21).

One study on high-protein drinks (325 mL) consumed within 15 min showed no differences in vomiting incidence or gastric emptying rates between the test and control groups (22). Compared to conventional diets, high-protein beverages have certain advantages in terms of nutritional value, absorption, portability, and preparation. High-protein drinks have been linked to a reduction in nausea by decreasing gastric arrhythmias, a benefit also observed in cancer patients undergoing chemotherapy (23). Additionally, patient satisfaction was higher in the high-protein drink group, with no change in neonatal APGAR scores (22).

Sodium bicarbonate (200 mL mixed with 4.26 g of sodium bicarbonate) was another intervention assessed. This intake was associated with a reduction in labor duration and instrumental delivery. In sports medicine, oral sodium bicarbonate was used to improve muscle function by buffering lactic acid (16). And it was similarly found to reduce uterine muscle fatigue and increase spontaneous delivery rates during labor (24).

Two studies found that parturients allowed to intake 200 mL carbohydrate beverage during labor had a higher rate of normal vaginal deliveries and a lower incidence of intrapartum complications compared to those restricted to ice chips only (25). This may be explained by the increased comfort and autonomy that carbohydrate-rich beverage intake provides, which subsequently reduces stress and labor complications (26).

Six other studies allowed parturients to consume ad libitum solid and liquid foods, not limited to specific foods, such as fruits, soups, and carbohydrate snacks like toast, fruits, and crackers. These interventions did not lead to any undesirable maternal or neonatal outcomes (17, 27–31).

### 3.4 Maternal and neonatal outcomes

The outcomes of the studies were categorized into five aspects. (a) Relate to duration of labor (time until full dilation, duration of the second stage, incidence of dystocia). (b) Relate to mode of assisted delivery (instrumental vaginal delivery, cesarean section, or oxytocin requirements). (c) Relate to gastrointestinal complications (incidence of vomiting or rate of gastric emptying). (d) Relate to maternal perception of labor (satisfaction score, anxiety, and pain levels). (e) Parturient ketosis (low blood glucose leading to ketosis); (f) Relate to neonatal outcomes (APGAR scores, stillbirths, venous–arterial lactate difference in the umbilical cord, neonatal deaths, NICU admissions, neonatal hypoglycemia). Regarding the duration of labor, most studies found either no significant differences between the groups or reported shorter labor durations in the test groups, indicating that appropriate oral intake during labor did not prolong labor duration. In terms of mode of assisted delivery, most studies found no differences between groups or noted a lower cesarean section rate in the test groups. For gastrointestinal complications, six studies found no differences between groups. Only one study showed more vomiting in the test group, potentially linked to the higher carbohydrate content of the apple or grape juice (14). Regarding maternal perception of labor, except for three studies found no differences in maternal satisfaction between the groups, one study showed lower subjective hunger score in the test group due to carbohydrate-rich beverage (26), another one study reported higher satisfaction scores in the test group due to the high-protein drink (22). Finally, this review reported no significant differences in maternal ketone levels and neonatal outcomes between groups.

## 4 Discussion

### 4.1 Main finding

We conducted a scoping review to map the published literature and synthesize research evidence on oral intake management during labor. The review found that moderate eating did not prolong labor duration or affect the vaginal mode of delivery in low-risk women. A policy of fluid intake during labor had less impact on maternal and neonatal outcomes than solid food intake, including mode of delivery and APGAR scores (17). Oral intake during labor did not significantly alter gastric emptying time or the incidence of vomiting; approximately 6 out of 7 studies (86%) supported this finding (14–17, 19, 22, 29). Less restrictive intake, particularly of carbohydrate-rich beverages and high-protein drinks, could improve the childbirth experience (22, 26).

### 4.2 According to the outcome indicators, we found the importance of oral intake management during labor and the necessity of a multidisciplinary team

As noted earlier, we identified that the outcomes related to maternal and neonatal health are affected by oral intake during labor. These include the rate of cesarean delivery, instrumental vaginal delivery, gastrointestinal complications, ketosis, and APGAR scores. The participants in this review were healthy, low-risk laboring women without maternal illnesses, and oral intake did not prolong labor duration. For most women with relatively short labors, a continuous supply of carbohydrates or fluids likely had little impact on maternal or neonatal outcomes (32). However, if labor becomes prolonged and intense, energy supplementation could prove beneficial (30). The uterus, one of the largest muscles in the human body, requires coordinated and effective contractions during labor, which demands significant energy (24). The laboring woman requested between 2,900 kJ and 3,600 kJ during a 9-h labor (18). Especially in the second stage, the energy requirements were greater (33). In a study of Chinese women, online oxygen uptake measurements during the first stage of labor were significantly lower (330 ± 0.2 kJ/h) compared to the second stage (464 ± 0.4 kJ/h) (18). Oral caloric intake during labor could prevent maternal hypoglycemia and ketoacidosis (18). Thus it’s important to concentrate on oral intake management during labor.

In this review, the juice without pulp containing higher carbohydrate intake might cause more vomiting (14). For parturients potentially requiring an epidural analgesia, oral intake during labor might reduce the pain at the expense of gastrointestinal complications such as nausea and vomiting. Due to the fear of aspiration during possible anesthesia, some places had attempted to minimize the risk of aspiration by restricting the oral intake in laboring women to ice chips/water or fasting to ensure an empty stomach since the 1940s (22). This concern stems from “Mendelson’s syndrome,” which describes pneumonitis resulting from aspiration of gastric contents, a condition associated with hypoxia, cyanosis, dyspnea, fever, pulmonary edema, and, in severe cases, death (34). However, modern techniques have successfully reduced the rates of regurgitation and aspiration pneumonia, mitigating the risks of Mendelson’s syndrome (30, 35). Besides, our study found that fluid intake during labor had less impact on obstetric or neonatal outcomes than solid intake, including on the mode of delivery and APGAR scores (17). Similarly, we did not observe any significant effects on neonatal outcomes, including APGAR scores, which aligns with previous research (35). This null finding might be explained by the smaller amounts of oral intake and the relatively short duration of labor, causing no venous–arterial difference yet in the umbilical cord (36). Through the outcomes of oral intake during labor, we found that food intake in labor had been restricted because of several aspects (37). Therefore, a multidisciplinary team—including anesthesiologists, obstetricians, and midwives—is essential for managing labor progression effectively (38).

### 4.3 Regarding the content of the oral intake intervention, we found the significance of informing parturients and providing personalized oral intake management

From this review, we found that oral intake management during labor varied widely across different cultural backgrounds and eating habits. For instance, ice chips were commonly used during labor in the United States but are not typically employed in China (28, 39). In contrast, date palm consumption was popular in the Middle East and in regions following Islamic Traditional Medicine (10). Although many organizations agreed that parturients could safely ingest food besides ice chips and water during labor, some foods had to be safer than others. As mentioned above, most interventions in test groups focused on carbohydrate intake, such as bread, biscuits, vegetables, fruits, low-fat yogurt, soups, fruit juice, and isotonic drinks. Other interventions included oral high-protein drinks, with one study even investigating sodium bicarbonate. Generally, light diet intake during labor has been found to prevent the rise of plasma beta-hydroxybutyrate and non-esterified fatty acids (30). Small amounts of carbohydrate intake did not increase the risk of prolonged labor, nor were they associated with an increased incidence of nausea or vomiting (17, 27–31). Early intake of richer carbohydrates during the second stage of labor, before full cervical dilation, had minimal impact on maternal and neonatal outcomes (12, 21). This aligns with findings from a meta-analysis by Ciardulli A et al. (35) who concluded that less-restrictive carbohydrate intake is associated with shorter labor duration and does not significantly increase vomiting incidence.

As labor progresses, contractions often suppress appetite. Previous research suggested that women tend to self-regulate their intake during the intrapartum period, preferring solids in early labor and switching to liquids as labor advances (40). About the time of oral intake, oral intake interventions were generally introduced during active labor when women are in the hospital, although many parturients in early labor might eat and drink freely at home. Some studies indicate that early oral carbohydrate intake does not significantly impact the incidence of labor dystocia (30). Therefore, it is crucial to inform parturients about the potential advantages and disadvantages of different types of oral intake during labor. This will allow them greater autonomy in their birth experience and improve their overall sense of control.

However, any oral intake decisions must be dynamically evaluated based on clinical situations and maternal preferences, with a focus on reassessing the risk of aspiration. As Sperling et al. suggested, low-residue foods (e.g., biscuits, toast, and cereals) should not be restricted for low-risk parturients, provided they understand the associated risks and give appropriate consent (41). Although it remained difficult to determine the optimal type of food or drink during labor, this review suggested that light carbohydrate intake is more appropriate in early labor, while high-protein drinks and isotonic energy drinks might be more suitable in the second stage of labor.

### 4.4 Strengths and limitations

This study had several strengths. It followed a rigorous and widely accepted review process to examine whether unrestricted oral intake during labor, compared to restricted intake, negatively affects maternal or neonatal outcomes (30).

However, several limitations should be noted. First, all participants were low-risk parturients without maternal illnesses. This may limit the generalizability of the findings, as these women might have been less susceptible to complications, potentially influencing maternal outcomes. Second, in some studies, oral intake was limited to specific “allowed” foods or drinks. Such restrictions may have affected women’s autonomy and influenced the outcomes of interest (22). Third, there was considerable heterogeneity in the control groups across studies—some allowed ice chips, others only water, placebo drinks, or complete fasting. Singata et al. argued that even withholding all food and fluids, or permitting only sips of water, constitutes an “intervention,” potentially affecting both clinical practice and study outcomes (32).

Additionally, several studies did not clearly address the impact of oral intake timing, fetal position, or fetal size on labor progression. These factors could influence labor duration but remain insufficiently explored. Therefore, future research should focus on identifying the most appropriate types and timing of oral intake during labor while considering evolving clinical circumstances and individual maternal needs.

## 5 Conclusion

In this scoping review, we found allowing restricted oral intake for low-risk women during labor does not appear to increase adverse maternal or neonatal outcomes and may help offset the fluid and energy loss associated with childbirth. However, given the potential risks such as aspiration and cesarean sections, careful consideration of both the content and timing of oral intake was essential. A light carbohydrate diet was more appropriate in early labor, while high-protein drinks and isotonic energy drinks might be more suitable in the second stage of labor. Providing individualized oral intake plans and counseling women on the benefits and risks of oral intake can enhance maternal satisfaction and support informed decision-making, thus continuous monitoring by a multidisciplinary team is crucial. This scoping review provides a reference for the design, research, and implementation of future oral intake management during labor.

## Data Availability

The original contributions presented in the study are included in the article/supplementary material, further inquiries can be directed to the corresponding author.

## Footnotes

^1^ https://osf.io/vmahc/overview
